# Changing epidemiology and antimicrobial susceptibility of bloodstream infections at a Vietnamese infectious diseases hospital (2010–2020)

**DOI:** 10.1038/s44259-024-00049-0

**Published:** 2024-10-16

**Authors:** Hoang Thu Trang Nguyen, Vinh Chau, Phu Huong Lan Nguyen, Hong Duc Du, Luong Nha Phuong Nguyen, Thi Quynh Ngan Le, Phuong Thao Huynh, Thi Nguyen To Nguyen, Thi Ngoc Dung Tran, Vinh Phat Voong, Thanh Tuyen Ha, Pham Nhu Quynh Nguyen, Stephen Baker, Guy Thwaites, Maia Rabaa, Duy Thanh Pham

**Affiliations:** 1 Oxford University Clinical Research Unit, Ho Chi Minh City, Vietnam; 2 Hospital for Tropical Diseases, Ho Chi Minh City, Vietnam; 3 Cambridge Institute of Therapeutic Immunology & Infectious Disease (CITIID) Department of Medicine, University of Cambridge, Cambridge, UK; 4 Centre for Tropical Medicine and Global Health, Nuffield Department of Medicine, University of Oxford, Oxford, UK

**Keywords:** Clinical microbiology, Antimicrobial resistance

## Abstract

Bloodstream infection (BSI) poses a global health problem, with diverse organisms and rising antimicrobial resistance (AMR). Here, we characterized trends in BSI prevalence, AMR, and antibiotic use at a Vietnamese infectious diseases hospital from 2010 to 2020. Among 108,303 cultured blood samples, 8.8% were positive, yielding 7995 pathogens. Of 7553 BSI cases, 86.4% were community-acquired. BSI prevalence varied from 17 to 35 cases/1000 admissions/year, highest in HIV/hepatitis wards and patients >60. The in-hospital mortality or hospice discharge outcome was 21.3%. The top three pathogens, *E. coli* (24%)*, K. pneumoniae* (8.7%) *and S. aureus* (8.5%) exhibited increasing prevalence and multidrug resistance. Pathogens like *Cryptococcus neoformans* (8.4%), *Talaromyces marneffei* (6.7%), and *Salmonella enterica* (6.5%) declined. *E. coli* and *K. pneumoniae* were prevalent in older adults with community-acquired BSIs. Antibiotic use reached 842.6 DOT/1000 PD and significantly reduced after an antibiotic control policy. Enhanced surveillance and antimicrobial stewardship are crucial for managing BSIs in Vietnam.

## Background

Bloodstream infection (BSI) is an important public health concern globally associated with significant morbidity and mortality^1–3^. The majority of BSI are attributed to bacterial pathogens with bacterial BSI ranking as a second most burdensome infectious disease symptom in 2019^4^. BSI is one of the most common severe infections in hospital settings, and nosocomial outbreaks with high mortality are frequently reported^5–7^. The causative agents of BSIs vary across geographical regions, hospital-onset and community-onset status and patient age^8^. Furthermore, the etiological distribution and the antimicrobial susceptibility patterns of BSIs change over time^9–12^, posing significant challenges for effective treatment, infection control and prevention measures.

While exhibiting geographical and demographic heterogeneity, *Escherichia coli, Salmonella enterica, Staphylococcus aureus*, and *Streptococcus pneumoniae* are among the most common causes of community-onset BSIs^1–3^. Since the turn of the 21st century, antimicrobial resistance (AMR) has emerged rapidly in these pathogens, particularly methicillin-resistant *S. aureus*^13^, ESBL-producing/carbapenem-resistant Enterobacterales^14^ and extensively drug-resistant *Salmonella* Typhi^15^. For hospital-acquired BSIs, the top causative agents vary by location but commonly include *S. aureus, E. coli, K. pneumoniae, Enterococcus spp., P. aeruginosa* and *A. baumannii*. These pathogens also display high resistance levels to many antimicrobials^2,8^.

In Vietnam, epidemiological surveillance of BSIs is limited, and existing data are often scattered and outdated, making it difficult to capture and predict the trends in disease etiology and AMR. Our previous BSI study at the Hospital for Tropical Diseases between 1994 and 2008 showed a declining frequency of *S*. Typhi BSI and an increasing frequency of HIV-associated pathogens, with modest increases in BSI caused by *E. coli* and *K. pneumoniae*^11^. Between 2011 and 2013, a study conducted at the National Hospital for Tropical Diseases in Hanoi, Vietnam, showed that *E. coli* and *K. pneumoniae* were the predominant causes of BSIs, associated with a high case fatality rate (34.7%). Data from an AMR surveillance of 13 hospitals in Vietnam (VINARES) also demonstrated that *E. coli* and *K. pneumoniae* were the two most common pathogens identified from blood and cerebrospinal fluid, exhibiting high proportions of multidrug resistance^16^. The causes and antimicrobial susceptibility of BSIs in Vietnam appear to be changing rapidly, which requires monitoring to improve treatment guidelines and inform public health interventions. In this study, we aimed to provide a comprehensive description of the clinical and microbiological characteristics, together with trends in antimicrobial susceptibility of BSI at a tertiary referral infectious diseases hospital in Vietnam from 2010 to 2020.

## Results

### Blood culture results

From 1 January 2010 to 31 December 2020, a total of 108,303 blood samples were submitted to the microbiology laboratory for culture, equivalent to ~10,000 blood cultures/year. The overall positivity rate for blood cultures was 8.8%. The annual positivity rate increased from 7.0% to 7.4% (years: 2010–2015) up to 8.9–14.1% (years: 2016–2020). In total, there were 9556 isolates from 9450 positive blood samples, with 8641 (88.8%) classified as recognized pathogens and 1095 (11.2%) as contaminants. The predominant contaminants were *Burkholderia cepacia* (35.9%) and coagulase-negative *Staphylococcus* (30.6%).

A total of 7995 non-duplicate BSI isolates were identified from 7775 blood samples, with 2.6% (203/7775) were classified as polymicrobial BSIs. There were 7553 culture-positive cases and 7733 BSI episodes, with 98.0% of patients experiencing BSI once and 2% having more than one BSI episode. Among these, 86.4% (6523/7553) were classified as CA-BSI, 12.7% (961/7553) as HA-BSI and 0.9% (69/7553) had missing information (Table 1).

**Table 1:** Characteristics of patients with bloodstream infections

	All data	CA-BSI^a,b^	HA-BSI^a,b^	p-value
Demographic	7553 patients	6523 patients	961 patients	
Age (years), median (IQR)	42.0 (31.0, 57.0)	42.0 (31.0, 57.0)	45.0 (30.0, 59.0)	0.059^c^
Male, *n* (%)	4721 (62.5)	4038 (61.9)	637 (66.3)	0.009^d^
Clinical wards, *n* (%)				<0.001^d^
Non-ICU Adult wards	3344 (44.3)	3000 (46.0)	326 (33.9)	
HIV	2019 (26.7)	1868 (28.6)	139 (14.5)	
Adult-ICU	1350 (17.9)	967 (14.8)	351 (36.5)	
CNS-ICU	479 (6.3)	409 (6.3)	68 (7.1)	
Children wards	211 (2.8)	174 (2.7)	37 (3.9)	
Pediatric-ICU	150 (2.0)	105 (1.6)	40 (4.2)	
Medical conditions, *n* (%)				
HIV	1468 (19.4)	1342 (20.6)	119 (12.4)	<0.001^d^
Liver diseases	1198 (15.9)	936 (14.3)	271 (28.2)	<0.001^d^
Others	4959 (65.7)	4314 (66.1)	587 (61.1)	0.002^d^
Outcome^e^	7308	6367	941	<0.001^d^
Good	4806 (65.8)	4254 (66.8)	552 (58.7)	
Poor	1560 (21.3)	1263 (19.8)	297 (31.6)	
Unknown (transfer/self-discharge)	942 (12.9)	850 (13.4)	92 (9.8)	
Length of stay (days), median (IQR)	13.0 (8.0, 19.0)	12.0 (7.0, 17.0)	26.0 (15.0, 40.0)	<0.001^c^

^a^CA-BSI: Community-acquired infection. HA-BSI: Hospital-acquired infection.

^b^There is a lack of information regarding the place of acquisition for 69 patients.

^c^Wilcoxon rank sum tes.

^d^Pearson’s Chi-squared test.

^e^There is missing outcome data for 245 patients.

### Prevalence of bloodstream infections

Between 2010 and 2020, 399,795 patients were admitted to HTD, 47.1% of whom were from the non-ICU adult wards, followed by the children wards (39.4%), ICU ward (8.3%) and HIV ward (5.2%). The overall BSI prevalence rate was 18.9 cases/1000 admission/year (Supplementary Table 1). The trend of BSI prevalence was upward, increasing from 17.4 cases/1000 admission in 2010 up to 35.2 cases/1000 admission in 2020 (*Z* = 2.49*, p* = 0.006). Overall, the highest BSI prevalence was observed in HIV ward (96.0 cases/1000 admission/year), followed by ICU wards (59.7 cases/1000 admission/year), adult infection ward B (41.5 cases/1000 admission/year), adult infection ward A (33.2 cases/1000 admission/year) and lowest in the children wards (1.3 cases/1000 admission/year) (Supplementary Table 2). The BSI prevalence displayed significantly increasing trend in adult infection ward B (*Z* = 3.74*, p* < 0.001) and adult infection ward A (*Z* = 2.96*, p* = 0.002) (Supplementary Table 3). The infection ward B specializes in treating patients with prolonged fever while the infection ward A is dedicated for patients with liver diseases.

### Demographic characteristics of bloodstream infection patients

The median age of all patients with BSI (*n* = 7553) was 42 years old (IQR: 31–57 years) (Table 1). The majority of patients (76.0%) aged between 15 and 60 years, while 19.3% aged >60 years and only 4.7% aged <15 years. The median age of patients from the HIV ward was 33 years old (IQR: 29–38 years), which was younger than those residing in other adult wards (median: 51 years, IQR: 38–62 years) (*p* < 0.001). There were significantly more male BSI patients than female BSI patients (62.5% vs. 37.5%, *p* < 0.001). This trend was largely mirrored in patients from HIV ward and CNS-ICU ward, of which 75.4% (1522/2019) and 67.9% (325/479) of patients were male, respectively. Conversely, there were more females than males in patients aged >60 years (59% vs. 41%, *p* < 0.001).

### Clinical characteristics of bloodstream infection patients

In our studied population, the most common underlying conditions among patients with BSI were HIV infection (19.4%) and acute/chronic liver diseases such as hepatitis/cirrhosis (16.0%) (Table 1). Saliently, between 2010 and 2020, the predominant underlying conditions of patients shifted from HIV infection towards liver diseases. Specifically, the prevalence of HIV infection among patients with BSI declined from 36.6% (243/664) in 2010 to 16.2% (118/730) in 2020 (*Z* = −1.7*, p* = 0.04). In contrast, the prevalence of liver diseases among patients with BSI increased steadily from 8.7% (58/664) in 2010 up to 23.9% (175/730) in 2020 (*Z* = 2.8*, p* = 0.003).

Outcome data were available for 7308 out of 7553 patients with BSI (96.8%). 4806 patients (65.8%) were discharged with a good outcome, whereas 1560 patients experienced a poor outcome. Additionally, 942 patients (12.9%) were discharged with unknown outcome (Table 1). The poor outcome rate was 21.3% (1560/7308), including 2.1% (154/7308) who died in the hospital and 19.2% (1406/7308) who were recorded as discharged home to die. The prevalence of poor outcome was significantly higher in HA-BSI (31.6%, 297/961) in comparison with CA-BSI group (19.8%, 1263/6523) (*p* < 0.001) (Table 1). The occurrence of poor outcome was highest in the HIV ward (33.7%), followed by ICU wards (32.3%) and lowest in the non-ICU adult wards (9.1%). Overall, the outcome of patients with BSI did not change significantly throughout the study period (*Z* = −0.47*, p* = 0.64).

### Pathogens causing bloodstream infections

Bacterial and fungal pathogens accounted for 84% (6719/7995 isolates) and 16% (1275/7995 isolates) of total isolates, respectively. *E. coli, K. pneumoniae and S. aureus* were the top three bacterial pathogens accounting for 24%, 8.7% and 8.5% of the isolates, respectively. Other major agents of BSIs were the two fungal pathogens, *Cryptococcus neoformans* (8.4%) and *Talaromyces marneffei* (6.7%). Other bacterial causes included *Salmonella enterica* (6.5%), *Streptococcus suis* (3.4%), *S. pneumoniae* (3.0%), *Burkholderia pseudomallei* (2.4%), *Aeromonas hydrophila* (1.9%) and *Stenotrophomonas maltophilia* (1.4%). The two common non-fermenting Gram-negative bacilli, *A. baumannii* and *P. aeruginosa*, each accounted for 1.7% of the isolates (Table 2).

**Table 2:** Causative agents of bloodstream infections

	Overall	CA-BSI	HA-BSI	*p* value
No blood samples	*N* = 7775	*N* = 6567	*N* = 1138	
Polymicrobial, *n*/tot^b^ (%)	203/7775 (2.6)	141/6567 (2.1)	58/1138 (5.1)	<0.001^a^
No of isolates	*N* = 7995	*N* = 6720	*N* = 1200	
Type of isolates, *n* (%)				
Gram-negative	4599 (57.5)	3787 (56.4)	764 (63.7)	
Gram-positive	2120 (26.5)	1763 (26.2)	342 (28.5)	
Fungi	1275 (15.9)	1169 (17.4)	94 (7.8)	
Leading causative agents (in order of prevalence), *n* (%)				
*Escherichia coli*	1915 (24.0)	1710 (25.4)	183 (15.3)	
*Klebsiella pneumoniae*	696 (8.7)	558 (8.3)	133 (11.1)	
*Staphylococcus aureus*	681 (8.5)	553 (8.2)	123 (10.3)	
*Cryptococcus neoformans*	671 (8.4)	643 (9.6)	21 (1.8)	
*Talaromyces marneffei*	532 (6.7)	500 (7.4)	29 (2.4)	
*Salmonella enterica*	523 (6.5)	481 (7.2)	39 (3.3)	
*Streptococcus suis*	273 (3.4)	268 (4.0)	3 (0.3)	
*Streptococcus pneumoniae*	238 (3.0)	221 (3.3)	16 (1.3)	
*Burkholderia pseudomallei*	189 (2.4)	171 (2.5)	16 (1.3)	
*Aeromonas hydrophila*	153 (1.9)	129 (1.9)	23 (1.9)	
*Burkholderia cepacia*	138 (1.7)	115 (1.7)	23 (1.9)	
*Pseudomonas aeruginosa*	135 (1.7)	72 (1.1)	60 (5.0)	
*Acinetobacter baumannii*	133 (1.7)	41 (0.6)	92 (7.7)	
*Stenotrophomonas maltophilia*	109 (1.4)	73 (1.1)	35 (2.4)	

^a^Pearson’s Chi-squared test.

^b^n = the number of polymicrobial blood cultures; tot = the total of blood cultures.

### Distribution of BSI pathogens by time and place of acquisition

The prevalence of the top three BSI agents: *E. coli* (*Z* = 2.65*, p* = 0.008), *K. pneumoniae* (*Z* = 3.27*, p* = 0.001), *S. aureus* (*Z* = 2.96*, p* = 0.003) increased significantly over time. Specifically, the prevalence of *E. coli*, *K. pneunoniae* and *S. aureus* rose from 9.2, 5.3 and 4.4 per 1000 blood cultures in 2010 up to 21.4, 10.1 and 8.0 per 1000 blood cultures in 2020, respectively. In contrast, the occurrence of HIV-associated pathogens such as *Talaromyces marneffei* (*Z* = −2.02*, p* = 0.04) exhibited a declining trend. The prevalence of other pathogens did not vary significantly during the study period (Supplementary Fig. 1).

*E. coli*, *Cryptococcus neoformans*, *Talaromyces marneffei*, *Salmonella enterica*, *Streptococcus suis*, *S. pneumoniae* and *Burkholderia pseudomallei* were the most common pathogens identified from patients with CA-BSIs. In contrast, HA-BSIs were predominantly associated with *S. aureus*, *Stenotrophomonas maltophilia*, *P. aeruginosa* and *A. baumannii* (Table 2).

### Distribution of BSI pathogens by underlying medical conditions and age

Among the patients with BSI who had liver diseases (*n* = 1,198), *E. coli*, *K. pneumoniae*, and *Aeromonas hydrophila* were the three predominant pathogens, constituting 33.5%, 18% and 7.8% of the cases, respectively. Notably, some minor pathogens (accounting for <0.5% of the isolates) were overrepresented in patients with BSI and concurrent liver diseases. For instances, 65.3% (32/49) of *Vibrio spp*., 62% (31/50) of *Campylobacter spp*., and 72% (36/50) of *Streptococcus salivarius* isolates were found in this particular patient group. Among the patients with BSI who had HIV infection (*n* = 1468), the prevalent pathogens were *Cryptococcus neoformans* (26.1%) and *Talaromyces marneffei* (22.8%), followed by *Salmonella enterica* (11.6%), *S. aureus* (10%) and *E. coli* (9.3%).

**Fig. 1:**
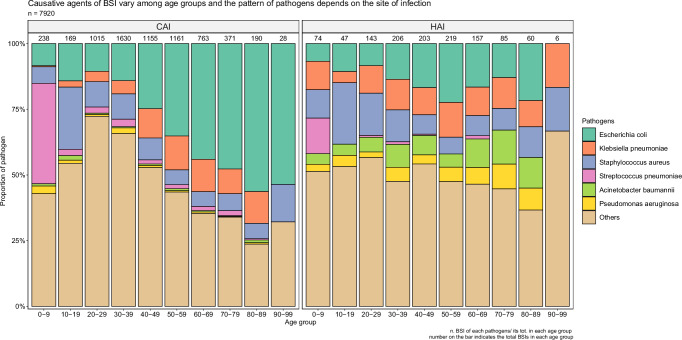
Distribution of bloodstream infection causes according to age and place of acquisition. The numerical value above each bar represents the total number of patients with bloodstream infections in each respective age group. The colored boxes indicates the proportion of bloodstream infection pathogens in each respective age group, with CAI representing community-acquired bloodstream infection and HAI representing hospital-acquired bloodstream infection.

There was a strong association between BSI pathogen and age among patients with CA-BSI (Fig. 1). This trend was particularly observed in *E. coli*, for which every 10-year increase in age, the prevalence of *E. coli* CA-BSI increased by 5.9%, (*p* < 0.001, linear regression) (Supplementary Fig. 2). Similarly, for every 10-year increase in age, the prevalence of *K. pneumoniae* CA-BSI increased by 1.5% (*p* = 0.002, linear regression) (Supplementary Fig. 2). The median age of patients infected with *E. coli* and *K. pneumoniae* were 54 (IQR: 41–65) and 51 (IQR: 39–61) years old, respectively. *Cryptococcus neoformans, Talaromyces marneffei* and *Salmonella enterica* were predominantly found in HIV patients with a median age of 33 (IQR: 29–38 years). For Gram-positive pathogens, *Streptococcus suis* was commonly detected in adults with a median age of 50 (IQR: 41–57 years), whereas *S. pneumoniae* was predominantly identified in young patients with a median age of 25 (IQR: 2–39 years).

### Antimicrobial resistance patterns of significant BSI pathogens

Here, we described the AMR patterns and trends for the top five bacterial pathogens causing BSI including *E. coli*, *K. pneumoniae*, *S. aureus*, *A. baumannii* and *P. aeruginosa*. The AMR prevalence was calculated based on the number of tested isolates for each clinically relevant antimicrobial (Table 3).

**Table 3:** Antimicrobial resistance patterns of critical pathogens of bloodstream infections

	*E. coli*	*K. pneumoniae*	*A. baumannii*	*P. aeruginosa*	*S. aureus*
No. isolates	1915	696	133	135	681
No. isolates with AST^a^	1820	643	123	121	640
MDR^b^	49.5 (901/1820)	15.6 (100/643)	46.3 (57/123)	18.2 (22/121)	75.6 (484/640)
MDR CA-BSI^c^	48.4 (789/1631)	10.7 (55/516)	15.3 (6/39)	4.8 (3/63)	73.9 (383/518)
MDR HA-BSI^c^	61.4 (105/171)	36.9 (45/122)	60.7 (51/84)	34.5 (19/55)	82.4 (98/119)
Gentamicin^d^	30.6 (232/758)	11.4 (31/272)	32.8 (19/58)	14.3 (16/112)	-^e^
Amikacin	1.2 (13/1102)	1.9 (8/414)	31.1 (32/103)	19.3 (23/119)	-
Piperacillin/tazobactam	6.3 (114/1815)	7.5 (48/640)	43.9 (54/123)	6.8 (8/118)	-
Ceftriaxone	56.1 (1013/1805)	14.4 (91/633)	-	-	-
Ceftazidime	54.8 (805/1470)	16 (83/519)	49.6 (60/121)	11.6 (14/121)	-
Cefepime	47.4% (853/1799)	12.6 (79/628)	49.2 (60/122)	19.5 (23/118)	-
Ertapenem	1.2 (22/1791)	4.4 (27/619)	-	-	-
Imipenem	0.6 (11/1794)	3.8 (24/629)	42.3 (52/123)	11.7 (14/120)	-
Meropenem	0.6 (10/1630)	4.7 (27/576)	43.9% (54/123)	11.8 (14/119)	-
Colistin	0 (0/26)	11.1 (3/27)	0.8 (1/118)	0 (0/78)	-
Trimethoprim/sulfamethoxazole	64.5 (1048/1624)	25.9 (156/602)	38.2 (42/110)	-	16.1 (79/492)
Ciprofloxacin	52.4% (676/1290)	22.2 (101/455)	39 (32/82)	20.4 (19/93)	37.8 (235/621)
Levofloxacin	52.6% (545/1036)	12.8 (46/359)	42.7 (44/103)	20.6 (22/107)	32.8 (171/521)
Moxifloxacin	-	-	-	-	26 (26/100)
Benzylpenicillin	-	-	-	-	98 (623/636)
Oxacillin	-	-	-	-	58.2 (370/636)
Erythromycin	-	-	-	-	71.9 (447/622)
Clindamycin	-	-	-	-	69.6 (436/626)
Rifampicin	-	-	-	-	3.8 (24/637)
Vancomycin	-	-	-	-	0 (0/633)
Teicoplanin	-	-	-	-	0.4 (1/262)
Linezolid	-	-	-	-	0 (0/288)

^a^AST (antimicrobial susceptibility test).

^b^MDR (Multidrug Resistance), % MDR (number of MDR isolates / total number of isolates tested).

^c^ MDR CA-BSI (Multidrug resistant community-acquired bloodstream infection), MDR HA-BSI (Multidrug resistant hospital-acquired bloodstream infection).

^d^% resistance (number of resistant isolates / total number of isolates tested).

^e^There were no antimicrobial susceptibility tests applied.

### AMR pattern and trend in *E. coli*

AMR pattern was available for 1820 out of 1915 *E. coli* isolates. The resistance proportions were 56.4% for 3rd/4th generation cephalosporins, 52.4% for fluoroquinolones (ciprofloxacin and/or levofloxacin), 13.7% for aminoglycosides, and 6.3% for piperacillin/tazobactam. The prevalence of carbapenem resistance was 1.5% (27/1807); the MIC50 and MIC90 for imipenem were equal at 0.025 µg/mL (*n* = 804). None of the tested *E. coli* isolates were resistant to colistin (Table 3). Only 12.6% of *E. coli* isolates were susceptible to all antibiotics tested. The prevalence of MDR *E. coli* was 49.5% (901/1820), exhibiting a lower occurrence in CA-BSI compared to HA-BSI (48.4%, 789/1631 vs. 61.4%, 105/171, *p* < 0.001) (Table 3).

**Fig. 2:**
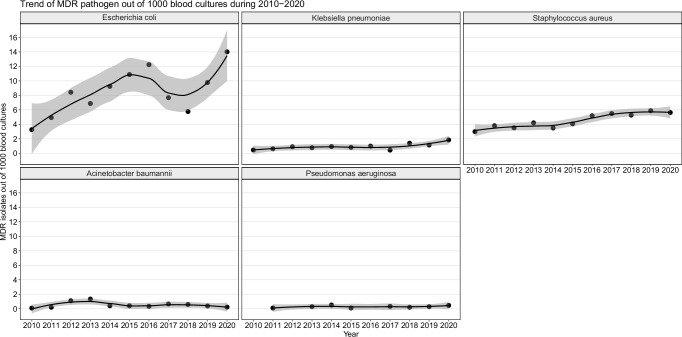
Trend of key multidrug resistant bacterial pathogens of bloodstream infections between 2010 and 2020. Each dot represents the number of multidrug resistant isolates out of 1000 blood cultures for each year. A Loess smoothing function creates a smoothed curve through the data points and the shaded area indicates the standard error associated with the curve.

**Fig. 3:**
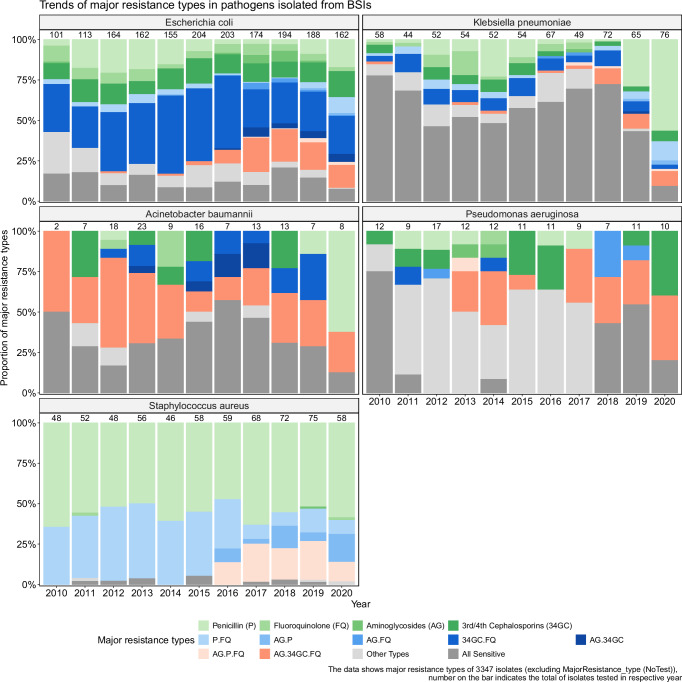
Trend of antimicrobial resistance patterns in major bacterial pathogens of bloodstream infections. The numbers above each bar indicate the total isolates identified for each year, while colored boxes show the proportions of major antimicrobial resistance types. P penicillins, FQ fluoroquinolones, AG aminoglycosides, 34GC 3rd/4th generation cephalosporins.

The prevalence of MDR *E. coli* followed an upward trend, increasing from 3.3 per 1000 blood cultures in 2010 to 14.0 per 1000 blood cultures in 2020 (*Z* = 2.2*, p* = 0.03) (Fig. 2). Additionally, MDR *E. coli* demonstrated an association with patient age, with a 2.3% increase in prevalence for every 10-year increase in age (linear regression*, p* < 0.001) (Supplementary Fig. 3). Notably, 33.6% of *E. coli* isolates were resistant to 3rd/4th generation cephalosporins and fluoroquinolones, while 10.9% were resistant to all three crucial antimicrobial classes- 3rd/4th generation cephalosporins, fluoroquinolones, and aminoglycosides. Alarmingly, resistance against all three classes surged from 1.7% (2010–2015) to 16.9% (2016–2020) (*p* < 0.001) (Fig. 3).

### AMR pattern and trend in *K. pneumoniae*

For *K. pneumoniae* BSI isolates (*n* = 643), the resistance frequencies to 3rd/4th generation cephalosporins, fluoroquinolones, aminoglycosides were 15.7%, 19.1% and 5.4%, respectively. The proportion of carbapenem resistance was 5.1% significantly higher than that of *E. coli* (1.5%) (*p* < 0.001). The MIC50 and MIC90 for imipenem were 0.25 µg/mL and 2 µg/mL (*n* = 278). Approximately 53.7% of *K. pneumoniae* isolates were susceptible to all antibiotics tested (Fig. 3).

The prevalence of MDR *K. pneumoniae* was 15.6%, with a threefold increase in HA-BSI (36.9%, 45/112) compared to CA-BSI group (10.7%, 55/516) (*p* < 0.001) (Table 3). The MDR *K. pneumoniae* increased from 0.5 per 1000 blood cultures in 2010 to 1.8 per 1000 blood cultures in 2020 (*Z* = 2.3*, p* = 0.02) (Fig. 2). Akin to *E. coli*, resistance to the three crucial antimicrobial classes rose from 1.8% (2010–2015) to 7.6% (2016–2020) (*p* = 0.0006) (Fig. 3). Notably, three *K. pneumoniae* isolates exhibited extensive resistance to all treatment drugs including carbapenems, colistin, levofloxacin, amikacin and tigecycline.

### AMR patterns and trends in *Staphylococcus aureus*, *A. baumannii*, *P. aeruginosa*

The resistance proportions of BSI *S. aureus* to oxacillin, fluoroquinolones and trimethoprim/sulfamethoxazole were 58.2 (370/636), 31.8% (196/616) and 16.1% (79/492), respectively. All *S. aureus* isolates were sensitive to vancomycin and linezolid. Only one *S. aureus* isolate was resistant to teicoplanin (0.4%, 1/262). The prevalence of MRSA and MDR *S. aureus* were 57.8% (370/640) and 75.6% (484/640), respectively. The prevalence of MDR *S. aureus* increased from 2.9 per 1000 blood cultures in 2010 to 5.6 per 1000 blood cultures in 2020 (Z = 3.1, *p* = 0.002) (Fig. 2). Interestingly, the dominant resistance pattern shifted from penicillins+fluoroquinolones (40%, 126/308) between 2010 and 2015 to penicillins+fluoroquinolones+aminoglycosides (19%, 63/332) and penicillins+aminoglycosides (9.3%, 31/332) between 2016 and 2020 (Fig. 3).

The resistance proportions of *A. baumannii* to the clinically used antibiotics were 51.2% for cephalosporins, 46.2% for fluoroquinolones, 36.6% for aminoglycosides, and 43.9% for carbapenems. The overall MDR rate of *A. baumannii* was 46.3% (57/123) and fluctuated during the study period (Fig. 2). Approximately one third (32.5%, 40/123) of *A. baumannii* isolates were sensitive to all tested antibiotics (Fig. 3). Only one isolate exhibited resistance to colistin (Table 3).

The resistance proportions of *P. aeruginosa* to commonly used antibiotics including piperacillin/tazobactam, ceftazidime and carbapenems were 6.8%, 11.6% and 11.7%, respectively. The prevalence of carbapenem resistance in *P. aeruginosa* was lower than in *A. baumannii* (*p* < 0.001). A proportion of 18.2% (22/121) *P. aeruginosa* isolates were sensitive to all tested antibiotics. The overall MDR prevalence was 18.2% (22/121) (Table 3).

### Antibiotic use for the treatment of bloodstream infections

**Fig. 4:**
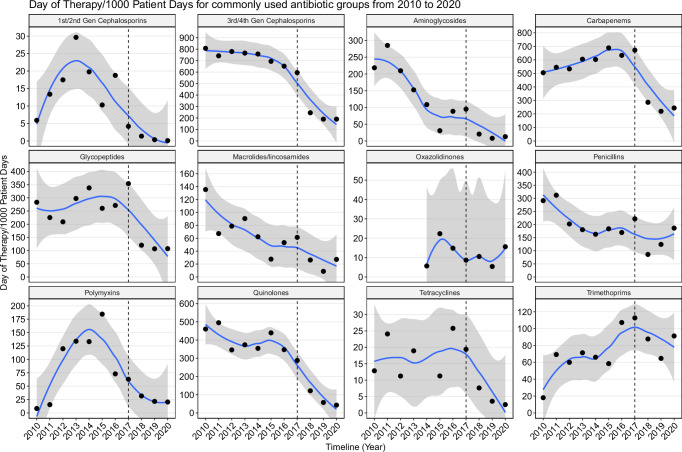
Trend of antibiotic consumption (Day of Therapy/1000 Patient Days) of commonly used antibiotic groups between 2010 and 2020. Each dot represents the Day of Therapy per 1000 Patient Day for each antibiotic in each year. A Loess smoothing function creates a smoothed curve through the data points and the shaded area indicates the standard error associated with the curve. The dashed line separates the periods before (2010–2016) and after (2017–2020) the implementation of the antibiotic control policy.

The antibiotic use was described for the patients with BSI from whom at least one recognized bacterial pathogen was identified and with available antibiotic prescription data (*n* = 4167 patients). Overall, the total amount of antibiotic consumption was 842.6 DOT/1000 PD. Between 2010 and 2016, the average antibiotic consumption was 970.8 DOT/1000 PD, exceeding the latter period 2017–2020 (760.9 DOT/1000 PD). The most frequently used antibiotics calculated as DOT/1000 PD at HTD were ceftriaxone (554.2), imipenem/cilastatin (339.2), vancomycin (227), levofloxacin (218.4), meropenem (172.1), ertapenem (140.6), amikacin (96.6), oxacillin (83.3), trimethoprim/sulfamethoxazole (73.3), colistin (73.2), and piperacillin/tazobactam (70.3) (Table 4). Following the implementation of antibiotic control policy, many antibiotics exhibited a significant decline in use, including 1st/2nd generation cephalosporins (cefuroxime*, p* = 0.018), 3rd/4th generation cephalosporins (ceftriaxone*, p* = 0.011; ceftazidime, *p* = 0.018), aminoglycosides (amikacin, *p* = 0.03), carbapenems (imipenem/cilastatin, *p* = 0.011), glycopeptides (teicoplanin, *p* = 0.02), macrolides/lincosamides (azithromycin, *p* = 0.047), penicillins (oxacillin, *p* = 0.03; ampicillin, *p* = 0.025) and quinolones (levofloxacin, *p* = 0.011; norfloxacin, *p* = 0.011), aminoglycosides (amikacin, *p* = 0.03) (Table 4, Fig. 4). Additionally, considerable reduction was observed in the consumption of last-line antibiotics such as meropenem, ertapenem, vancomycin and colistin (Table 4). In 2020, the top antibiotics used for BSI treatment at HTD were ceftriaxone (169.3 DOT/1000 PD), imipenem/cilastatin (151.7 DOT/1000 PD), piperacillin/tazobactam (132.1 DOT/1000 PD) and vancomycin (104.4 DOT/1000 PD).

**Table 4:** Antibiotic consumption (Day of Therapy/1000 Patient Days) for bacterial bloodstream infections

Group Antibiotic	Characteristic	Overall^a^	2010–2016^a^	2017–2020^a^	*p* value^b^
1st/2nd Gen Cephalosporins	Cefuroxime	10.0 (0.1–27.9)	14.9 (1.4–27.9)	1.3 (0.1–4.2)	0.012
	Cefaclor	3.1 (1.7–4.5)	3.1 (1.7–4.5)	-	-
	Cefalexin	2.7 (0.8–4.6)	4.6 (4.6–4.6)	0.8 (0.8–0.8)	>0.9
3rd/4th Gen Cephalosporins	Ceftriaxone	554.2 (167.2–766.7)	705.9 (630.4–766.7)	288.8 (167.2–585.5)	0.006
	Ceftazidime	57.9 (9.5–157.2)	82.5 (23.9–157.2)	14.8 (9.5–24.7)	0.012
	Cefixime	11.0 (0.1–23.5)	12.8 (1.6–23.5)	0.1 (0.1–0.1)	0.3
	Cefoperazone/sulbactam	3.7 (0.7–6.7)	-	3.7 (0.7–6.7)	-
	Cefpodoxime	3.2 (0.1–9.3)	-	3.2 (0.1–9.3)	-
	Cefotaxime	1.6 (1.2–2.0)	-	1.6 (1.2–2.0)	-
	Ceftolozane/tazobactam	2.2 (2.2–2.2)	-	2.2 (2.2–2.2)	
	Cefepime	2.6 (0.4–6.1)	6.1 (6.1–6.1)	0.8 (0.4–1.2)	0.7
	Ceftazidime/avibactam	0.2 (0.2–0.2)	-	0.2 (0.2–0.2)	-
Aminoglycosides	Amikacin	96.6 (4.5–272.5)	139.0 (26.8–272.5)	22.3 (4.5–65.8)	0.024
	Gentamicin	20.8 (2.1–67.1)	20.2 (2.3–57.1)	21.9 (2.1–67.1)	0.6
	Streptomycin	6.3 (1.4–22.1)	8.8 (1.6–22.1)	1.5 (1.4–1.6)	0.3
Carbapenems	Imipenem/cilastatin	339.2 (116.1–545.3)	430.1 (361.5–545.3)	180.0 (116.1–327.7)	0.006
	Meropenem	192.9 (41.9–328.1)	235.7 (153.3–328.1)	133.1 (41.9–305.3)	0.11
	Ertapenem	172.1 (41.7–305.3)	199.1 (153.3–244.3)	124.7 (41.7–305.3)	**0.2**
Glycopeptides	Vancomycin	227.0 (102.2–350.1)	260.6 (208.3–322.0)	168.2 (102.2–350.1)	0.2
	Teicoplanin	15.9 (3.1–44.5)	25.4 (7.3–44.5)	4.0 (3.1–4.7)	0.016
Macrolides/ Lincosamides	Azithromycin	36.1 (3.2–74.3)	47.3 (21.6–74.3)	16.5 (3.2–40.1)	0.042
	Clindamycin	8.5 (1.2–15.7)	7.9 (1.2–15.7)	9.4 (4.7–14.3)	0.8
	Clarithromycin	8.2 (0.8–21.0)	10.5 (1.8–21.0)	4.8 (0.8–8.4)	0.3
	Erythromycin	10.1 (4.5–15.8)	10.1 (4.5–15.8)	-	
	Spiramycin	22.5 (0.8–56.6)	33.3 (10.1–56.6)	0.8 (0.8–0.8)	0.7
Oxazolidinones	Linezolid	11.9 (5.4–22.3)	14.3 (5.7–22.3)	10.1 (5.4–15.6)	0.6
Penicillins	Oxacillin	83.3 (18.7–181.9)	108.8 (53.0–181.9)	38.6 (18.7–80.5)	0.024
	Piperacillin/tazobactam	70.3 (29.2–132.1)	**-**	70.3 (29.2–132.1)	**-**
	Piperacillin	46.6 (2.4–92.7)	54.2 (29.0–92.7)	28.7 (2.4–77.8)	0.4
	Amoxicillin/clavulanic	21.6 (2.1–99.6)	33.8 (2.1–99.6)	3.3 (2.2–5.7)	0.2
	Ampicillin	18.0 (3.4–51.2)	25.0 (14.0–51.2)	7.5 (3.4–14.0)	0.019
	Benzylpenicillin	15.4 (0.4–49.3)	21.0 (5.0–49.3)	7.1 (0.4–17.9)	0.4
	Phenoxymethylpenicillin	2.2 (1.2–3.3)	3.3 (3.3–3.3)	1.2 (1.2–1.2)	>0.9
	Ampicillin/sulbactam	8.5 (1.4–12.3)	-	8.5 (1.4–12.3)	-
	Ticarcillin/clavulanic	4.1 (1.3–9.5)	5.4 (1.3–9.5)	1.4 (1.4–1.4)	>0.9
	Cloxacillin	1.9 (0.1–3.5)	3.5 (3.5–3.5)	1.1 (0.1–2.0)	0.7
Polymyxins	Colistin	73.2 (8.3–184.5)	95.4 (8.3–184.5)	34.1 (20.4–62.9)	0.3
Quinolones	Levofloxacin	218.4 (31.1–448.7)	292.9 (236.8–448.7)	88.1 (31.1–184.2)	0.006
	Norfloxacin	56.9 (1.2–157.9)	83.8 (36.6–157.9)	9.9 (1.2–27.5)	0.006
	Ciprofloxacin	43.2 (7.6–163.9)	47.3 (12.6–163.9)	35.9 (7.6–105.0)	0.3
	Ofloxacin	32.5 (6.4–65.4)	32.5 (6.4–65.4)	-	-
	Moxifloxacin	4.7 (0.5–14.9)	6.7 (2.1–14.9)	3.2 (0.5–6.8)	0.6
Tetracyclines	Doxycycline	13.6 (2.5–25.8)	17.4 (11.2–25.8)	8.0 (2.5–19.4)	0.11
	Tigecycline	1.1 (1.1–1.1)	-	1.1 (1.1–1.1)	-
Trimethoprims	Trimethoprim/sulfamethoxazole	73.3 (18.1–112.7)	64.3 (18.1–107.2)	89.1 (64.7–112.7)	0.2

^a^Mean (Min–Max).

^b^Wilcoxon rank sum exact test.

## Discussion

This study is a continuation of previous BSI surveillance at HTD from 1994 to 2008^11^, offering a unique opportunity to depict the disease epidemiological trends over nearly 30 years. Compared to the period of 1994–2008, we observed a prominent shift in the etiological distribution of BSIs during 2010–2020, with a declining trend of HIV-associated pathogens such as *Talaromyces marneffei* and *Salmonella enterica* and an increasing trend of Gram-negative pathogens such as *E. coli* and *K. pneumoniae*. Further, typhoid fever which had been shown to decline during 1994–2008 continued to diminish during 2010–2020. The reduction of HIV-associated BSI pathogens might stem from the country’s efforts in the detection, treatment and prevention of HIV^17^, contributing to the reported decrease in new HIV cases and deaths between 2011 and 2015^18^. The epidemiological drivers for the emergence of *E. coli* and *K. pneumoniae* as the main BSI causes are incompletely understood. Similar trend has also been reported in a multi-country surveillance^8^, UK^19^, France^20^, Canada^21^, Thailand^22^, China^23^. Previous studies have suggested that older age^24,25^, increasing prevalence of underlying health conditions^26,27^ and the spread of multiple pandemic clones of *E. coli* and *K. pneumoniae*^20^ are important factors contributing to the rise of Gram-negative BSI pathogens worldwide.

Here, we found a striking dominance of *E. coli* and *K. pneumoniae* among patients with community-acquired BSI, particularly those aged ≥ 60 years. Furthermore, a high proportion of patients with *E. coli* and *K. pneumoniae* BSI had liver diseases. *E. coli* bacteraemia has been widely reported to cause a substantial burden in older adults with increased incidence and mortality rates^21,24,25,28^. This trend is of particular concern as Vietnam is among the countries experiencing the highest rates of ageing during the last decade, in which the older population (aged ≥ 60 years) grew considerably from 7.45 million in 2009 to 11.41 million in 2019 with the annual growth rate of 4.35%, four time higher than the average rate of total population^29^. Given the rapid population ageing in Vietnam, the economic and health burdens of BSIs is likely to escalate in the future. Collectively, our findings suggest that changing population demographics and prevalence of underlying diseases are likely the major factors driving the etiological shift of BSIs in Vietnam. Routine surveillance is necessary to monitor the epidemiology, etiology, and AMR trends for improving treatment guidelines. As *E. coli* and *K. pneumoniae* are parts of the normal intestinal flora, understanding the mechanisms of gut colonization and translocation/progression from colonization to disease would aid the development of innovative countermeasures. In long term, global strategic investment on vaccine research and development with a focus on preventing BSIs caused by *E. coli* and *K. pneumoniae* is of urgent needs.

Between 2010 and 2020, we observed an increasing trend in the prevalence of MDR and resistance to empirical treatment drugs in the predominant BSI pathogens, including *E. coli, K. pneumoniae* and *S. aureus*. Our findings align with previous multiple hospital surveillance studies conducted in Vietnam between 2012–2013 and 2016–2017. These studies documented increasing frequencies of MDR and ceftriaxone-resistant *E. coli, K. pneumoniae*, and MRSA identified from blood and cerebrospinal fluid^16,30^. In our study, more than 30% of *E. coli* isolates were resistant to 3^rd^/4^th^ generation cephalosporins and fluoroquinolones, rendering these important drugs ineffective and leading to increased reliance on carbapenems. However, the continued reliance on carbapenems raise concerns about the inevitable rise of carbapenem resistance. Although the MDR prevalence of *K. pneumoniae* was lower but than that of *E. coli*, *K. pneumoniae* displayed greater resistance to last-resort antibiotics (carbapenems, colistin), posing a serious threat to the effectiveness of antimicrobial therapy. The rates of multidrug resistant and methicillin resistant *S. aureus* and carbapenem resistant *A. baumannii* appeared to increase annually.

In this study, we also reported the antibiotic consumption for the treatment of BSIs, providing insight into the correlation between AMR pattern and antibiotic use. Overall, the antibiotic consumption was notably high, reaching a total of 842.6 DOT/1000 PD. Particularly, there was substantial use of broad-spectrum antibiotics including ceftriaxone and carbapenems (>400 DOT/1000 PD), followed by vancomycin, levofloxacin (>150 DOT/1000 PD), trimethoprim/sulfamethoxazole, amikacin, colistin and oxacillin (>60 DOT/1000 PD). There were notable trends in the antibiotic consumption rates over the study period, including a decrease of ceftriaxone, levofloxacin, amikacin and oxacillin, concurrent with an increase of carbapenems and vancomycin between 2010 and 2016. These opposite trends are probably driven by the rise of ceftriaxone resistant and multidrug resistant Gram-negative bacteria and methicillin resistant *S. aureus* as described above. After the implementation of the antibiotic control policy, a substantial decrease in the prescription of most antibiotics, including commonly used and last-resort antibiotics, was evident. These findings indicate that antibiotics, especially those considered last-resort options, had been excessively utilized in the treatment of BSI. Moreover, they underscore the significant impact of antibiotic control policies in mitigating antibiotic overuse and promoting responsible prescribing practices.

Our study has some limitations. Due to the nature of retrospective study, we could not capture all the underlying diseases of patients and the sources of BSI or further investigate the high contamination rate of *Burkholderia cepacia*, a potential cause of BSI and nosocomial outbreaks. Outcomes were only observed during hospital stay and long-term consequences beyond hospital discharge was largely unknown. Pediatric BSIs are often treated at other hospitals and thus the etiological distribution might have not been fully captured in this study. Moreover, the stratification of hospital-acquired versus community-acquired BSI lacked supporting data regarding patient referral and prior healthcare exposure, potentially leading to misclassification of some HA-BSI cases as CA-BSI. Despite these limitations, our study provided up-to-date and systemic data of BSI in Vietnam across 11 years and uncovered factors associated with the changing epidemiology, etiology, AMR profile and antibiotic consumption. Our findings could facilitate the revision of current treatment guidelines and advocate for enhanced surveillance of BSI at a larger scale.

In conclusion, through a systemic surveillance BSIs at the largest referral infectious diseases hospital in southern Vietnam between 2010 and 2020, we uncovered the increasing trend and preponderance of the Gram-negative bacterial agents of *E. coli* and *K. pneumoniae* and the Gram-positive pathogen *S. aureus*. These organisms exhibited a high prevalence of MDR and resistance to commonly prescribed drugs, including 3rd/4th generation cephalosporins, fluoroquinolones, aminoglycosides and oxacillin. Antibiotic consumption for bacterial BSIs was notably extensive and spanned a wide range of antimicrobial drugs, in accordance with the rapid increase of AMR among the infecting organisms. Strengthened antibiotic control policy had a dramatic impact on the antibiotic prescription rates, which indicated the potential overuse of antibiotics including the last-resort drugs. The majority of BSIs occurred in the community and were associated with elderly people and those with HIV infections and liver diseases. Changing population demographics and underling medical comorbidities are likely the factors driving the shift in etiology, incidence and AMR emergence of BSIs. Continued surveillance and antibiotic stewardship are instrumental to guide appropriate treatment therapies. Furthermore, future research is needed to understand the risk factors for colonization/infection development and to develop vaccines and therapeutics against *E. coli* and *K. pneumoniae*.

## Methods

### Study design and population

We performed a retrospective, descriptive study including all positive blood isolates recovered from patients with BSI admitted to the Hospital for Tropical Diseases (HTD) between 1 January 2010 and 31 December 2020. Located in Ho Chi Minh City (HCMC), HTD is a 660-bed tertiary referral hospital for patients with infectious diseases in southern Vietnam (population >40 millions)^31^. HTD has 14 clinical wards, which include three intensive care units (ICUs) for adults, children, and central nervous system infection (referred to as CNS-ICU), six general adult wards, four general pediatric wards, and one HIV ward. The hospital provides healthcare services for >36,000 in-patients per year.

The hospital does not receive or manage neonates or patients without infectious diseases, including those with surgical requirements, pulmonary tuberculosis, cancer, or primary hematological or immunological disorders^32^.

### Data collection

Clinical, microbiological and antibiotic consumption data were collected from the hospital’s computerized databases. Microbiological data covered patient identification number, treatment ward, sample identification number, date of sampling, name of organism, and antimicrobial susceptibility data. Clinical data included gender, age, treatment ward, date of admission, date of discharge, underlying medical condition, discharge diagnosis and outcome. The clinical outcome was categorized as good (discharged with recovery), poor (death or discharged home to die) or unknown (patient transferred to another hospital or self-discharged). Patients with missing outcomes due to unavailable information in the hospital database were removed from the denominators of relevant calculations. The underlying medical conditions were categorized into HIV infection, liver diseases (hepatitis and cirrhosis), and others (i.e., diabetes, cardiovascular diseases, or unrecorded).

### Definitions

A BSI episode was defined as the isolation of at least one clinically relevant pathogen from one blood culture drawn from a patient with a clinical syndrome indicative of a BSI^33^. A new episode was recorded if a positive blood culture with the same organism was identified more than 14 days, or a different organism was identified more than two days after the first positive blood culture. When multiple organisms of the same species were identified within a BSI episode, only the first organism was included in the analyses.

Coryneform (Corynebacterium, etc.), Coagulase-negative Staphylococci (CoNS), Micrococci, Propionibacterium, Bacillus, alpha-hemolytic Streptococci, environmental Gram-negative Bacilli, and non-pathogenic Neisseria were regarded as contaminants unless isolated from two or more separate blood culture sets^34^. For every case of contaminant, a final decision was made based on the agreement between a microbiologist and an attending clinician.

Hospital-acquired BSI (HA-BSI) was defined as a positive blood culture obtained on day three or later from hospital admission. Community-acquired BSI (CA-BSI) was defined as a positive blood culture obtained on the day or less than three days of hospital admission. Polymicrobial BSI was defined as the isolation of more than one microorganism from the same blood culture.

Multidrug resistance (MDR) was defined as acquired non-susceptibility to at least one agent in three or more antimicrobial categories that are clinically relevant^35^. Here, the antimicrobial categories included aminoglycosides, penicillins with beta-lactamase inhibitors, 3rd/4th generation cephalosporins, carbapenems, fluoroquinolones, folate pathway inhibitors, polymyxins and glycylcycline.

### Blood culture and antimicrobial susceptibility testing

The BACTEC blood culture system (Becton Dickinson) was used for blood culture. An aliquot of 5–8 mL (for adults) or 2–5 mL (for children) of venous blood was inoculated into BACTEC plus aerobic bottles. Inoculated bottles were incubated at 37 ^°^C in a BACTEC 9050 automated analyser for up to five days and sub-cultured on fresh sheep blood agar, chocolate agar or Sabouraud’s agar (when yeast or mold was suspected) when the machine indicated a positive signal. Plates were incubated at 37 °C in the air (for blood agar), 5% CO_2_ (for chocolate agar) for two days or 30 °C in the air (for Sabouraud’s agar) for five days. Organisms were identified by standard methods, including API20E and API20EN identification kits (BioMérieux) or MALDI-TOF mass spectrometry (Bruker). Antimicrobial susceptibility testing (AST) was performed by VITEK automated machine or the disk diffusion method using guidelines established by the Clinical and Laboratory Standards Institute (CLSI). The AST results were interpreted according to the 2020 CLSI guidelines.

### Antibiotic consumption

Antibiotic consumption data covered drug name, dose and dates of start and stop of treatment for each antibiotic. In-hospital antibiotic consumption was measured as days of therapy (DOT) per 1000 patient-days (PD). One DOT represents the use of a single antibiotic on a given day. Moreover, antibiotic consumption was analyzed for the periods 2010–2016 and 2017–2020 to assess the impact of the antibiotic control policy issued by the Ministry of Health (MoH) on March 04, 2016. This policy outlined essential principles for the appropriate use of antibiotics as detailed in the “Instructions on management of the antibiotic use in hospitals”. These instructions include specific guidance on “use of antibiotics for bacterial infection only”, “appropriate antibiotic selection”, “appropriate dosage, route of administration and duration”, “appropriate combination therapy” and “appropriate antibiotic prophylaxis”. The Decision 772/QD-BYT also required every hospital to establish a formal antimicrobial stewardship team to monitor antibiotic use in the hospital and promote rational use. The Hospital for Tropical Diseases adhered to the overarching directives from the MoH and formulated their internal guidelines for the proper use of antibiotics, which were subsequently implemented in late 2016.

### Statistical analysis

Analyses were performed using statistical software R (version 4.2.2). Mean (standard deviation, SD) or median (interquartile range, IQR) were reported for continuous variables and comparisons between groups were performed using unpaired samples t-test or Wilcoxon rank sum test. Categorical variables were described using frequencies and percentages, with comparisons between groups performed using chi-square test or a Fisher’s exact test where appropriate. The *p* ≤ 0.05 were considered statistically significant. The time-series data were assessed using non-parametric Mann-Kendall test, for which a *p* ≤ 0.05 indicates a statistically significant trend. Additionally, a locally weighted regression (Loess) smoothing function was utilized to generate a smoothed curve through a set of data points. Linear regression was employed to assess the association between *E. coli*/*K. pneumoniae* BSI and patient age.

## Supplementary information

Supplemental Information

## Data Availability

The data supporting the findings of this study are not openly available due to sensitive reasons. However, they can be obtained from the corresponding author upon reasonable request. The data are stored in controlled access data storage at Oxford University Clinical Research Unit.
