# Supplementary material for: Changing epidemiology and antimicrobial susceptibility of bloodstream infections at a Vietnamese infectious diseases hospital (2010–2020)
Source: NPJ Antimicrob Resist. 2024 Oct 16;2:32. doi: 10.1038/s44259-024-00049-0 (PMC11485239; doi:10.1038/s44259-024-00049-0)
Supplement: Supplementary file 1 — Supplemental Information [file 44259_2024_49_MOESM1_ESM.pdf]

Supplementary Table 1. The prevalence of bloodstream infections per 1,000 admission between 2010 and 2020

Study year	BSI cases	Total hospitalized patients	BSI prevalence (BSI cases per 1,000 admission)
2010	664	38177	17.4
2011	604	38805	15.6
2012	639	41508	15.4
2013	660	36696	18
2014	609	37249	16.3
2015	633	39139	16.2
2016	731	38029	19.2
2017	730	34746	21
2018	814	35357	23
2019	739	39357	18.8
2020	730	20732	35.2
Overall	7553	399795	18.9

Supplementary Table 2. The prevalence of of bloodstream infections per 1,000 admission by clinical wards

Clinical wards	BSI cases	Total hospitalized patients	BSI prevalence (BSI cases per 1,000 admission)
**Children wards**	**211**	**157406**	**1.3**
Children ward A	46	45257	1
Children ward B	74	37806	2
Children ward C	28	34733	0.8
Children ward D	63	39610	1.6
**HIV**	**2019**	**21028**	**96**
**ICU**	**1979**	**33150**	**59.7**
CNS-ICU	479	8016	59.8
General-ICU	1350	15128	89.2
Paediatric-ICU	150	10006	15
**Non-ICU Adult wards**	**3344**	**188211**	**17.8**
Infection ward A	935	28146	33.2
Infection ward B	1124	27086	41.5
Infection ward C	311	37554	8.3
Infection ward D	174	33256	5.2
Internal ward A	224	33029	6.8
Internal ward B	576	29140	19.8

Supplementary Table 3. Time-trend analysis of bloodstream infection prevalence per 1000 admissions across different hospital wards

Clinical wards	2010	2011	2012	2013	2014	2015	2016	2017	2018	2019	2020	Mann-Kendall test
**Children wards**												
Children ward A	1.49 (6/4037)	1.7 (7/4123)	1.43 (7/4909)	2.27 (10/4406)	0.21 (1/4769)	0 (0/5127)	0.41 (2/4890)	1.56 (6/3849)	0.83 (3/3622)	0.5 (2/4023)	1.33 (2/1502)	Z=-0.78, p=0.4
Children ward B	1.68 (6/3579)	0.81 (3/3704)	0.68 (3/4414)	1.41 (5/3554)	2.16 (8/3700)	1.27 (5/3923)	1.94 (7/3607)	3.69 (12/3255)	3.64 (12/3300)	3.22 (10/3106)	1.8 (3/1664)	Z=1.71, p=0.087
Children ward C	2.67 (8/2997)	1.03 (4/3871)	0.94 (4/4237)	0 (0/3372)	0.55 (2/3662)	0 (0/3354)	0.64 (2/3107)	0.34 (1/2941)	0.34 (1/2907)	1.13 (3/2666)	1.85 (3/1619)	Z=-0.08, p>0.9
Children ward D	0.29 (1/3419)	0.28 (1/3577)	0.48 (2/4162)	4.1 (16/3905)	2.81 (12/4275)	1.35 (6/4438)	2 (9/4490)	1.7 (6/3538)	2.23 (7/3142)	0.59 (2/3397)	0.79 (1/1267)	Z=0.62, p=0.5
**HIV**	100.22 (270/2694)	83.14 (211/2538)	85.97 (193/2245)	85.82 (164/1911)	82.24 (144/1751)	71.81 (122/1699)	115.57 (170/1471)	104.28 (168/1611)	130.72 (223/1706)	100.06 (172/1719)	108.14 (182/1683)	Z=1.09, p=0.3
**ICUs**												
General-ICU	85.39 (135/1581)	92.8 (143/1541)	95.27 (159/1669)	99.88 (163/1632)	96.31 (94/976)	90.99 (101/1110)	92.11 (105/1140)	104.33 (106/1016)	79.79 (105/1316)	58.43 (104/1780)	98.76 (135/1367)	Z=0, p>0.9
Paediatric-ICU	12.56 (17/1353)	14.72 (19/1291)	11.1 (16/1441)	12.66 (19/1501)	14.61 (15/1027)	11.87 (9/758)	15.36 (12/781)	15.44 (8/518)	28.57 (15/525)	16.33 (9/551)	42.31 (11/260)	Z=2.8, p=0.005
CNS-ICU	80.49 (59/733)	69.01 (55/797)	25.11 (34/1354)	30.91 (46/1488)	31.51 (27/857)	84.49 (55/651)	94.2 (52/552)	81.59 (35/429)	103.96 (42/404)	87.56 (38/434)	113.56 (36/317)	Z=2.49, p=0.013
**Non-ICU Adult wards**												
Infection ward A	14.34 (43/2998)	15.88 (43/2708)	18.82 (51/2710)	23.98 (63/2627)	37.59 (98/2607)	47.51 (120/2526)	43.11 (113/2621)	41.7 (106/2542)	43.91 (114/2596)	40.57 (109/2687)	49.21 (75/1524)	Z=2.96, p=0.003
Infection ward B	16.31 (38/2330)	28.31 (65/2296)	34.65 (79/2280)	34.25 (72/2102)	43.18 (107/2478)	34.67 (100/2884)	47.6 (126/2647)	48.31 (114/2360)	53.23 (141/2649)	48.52 (152/3133)	67.46 (130/1927)	Z=3.74, p<0.001
Infection ward C	5.15 (19/3690)	3.37 (12/3558)	7.59 (27/3555)	10.75 (31/2885)	8.28 (25/3020)	9.72 (35/3601)	8.79 (33/3753)	8.07 (29/3593)	10.98 (39/3552)	7.64 (32/4188)	13.43 (29/2159)	Z=1.87, p=0.062
Infection ward D	8.4 (24/2858)	3.42 (11/3213)	4.67 (15/3210)	5.93 (15/2531)	9.65 (27/2799)	2.87 (9/3133)	3.42 (11/3215)	4.73 (16/3383)	5.68 (20/3523)	4.21 (19/4510)	7.95 (7/881)	Z=0, p>0.9
Internal ward A	3.57 (12/3360)	3.55 (11/3095)	6.85 (20/2919)	7.25 (19/2619)	2.29 (7/3051)	6.52 (21/3222)	7.87 (25/3177)	8.37 (25/2987)	6.49 (20/3080)	5.67 (19/3349)	20.74 (45/2170)	Z=1.4, p=0.2
Internal ward B	10.2 (26/2548)	7.62 (19/2493)	12.07 (29/2403)	17.11 (37/2163)	18.45 (42/2277)	18.43 (50/2713)	24.83 (64/2578)	35.98 (98/2724)	23.72 (72/3035)	17.83 (68/3814)	29.68 (71/2392)	Z=2.65, p=0.008

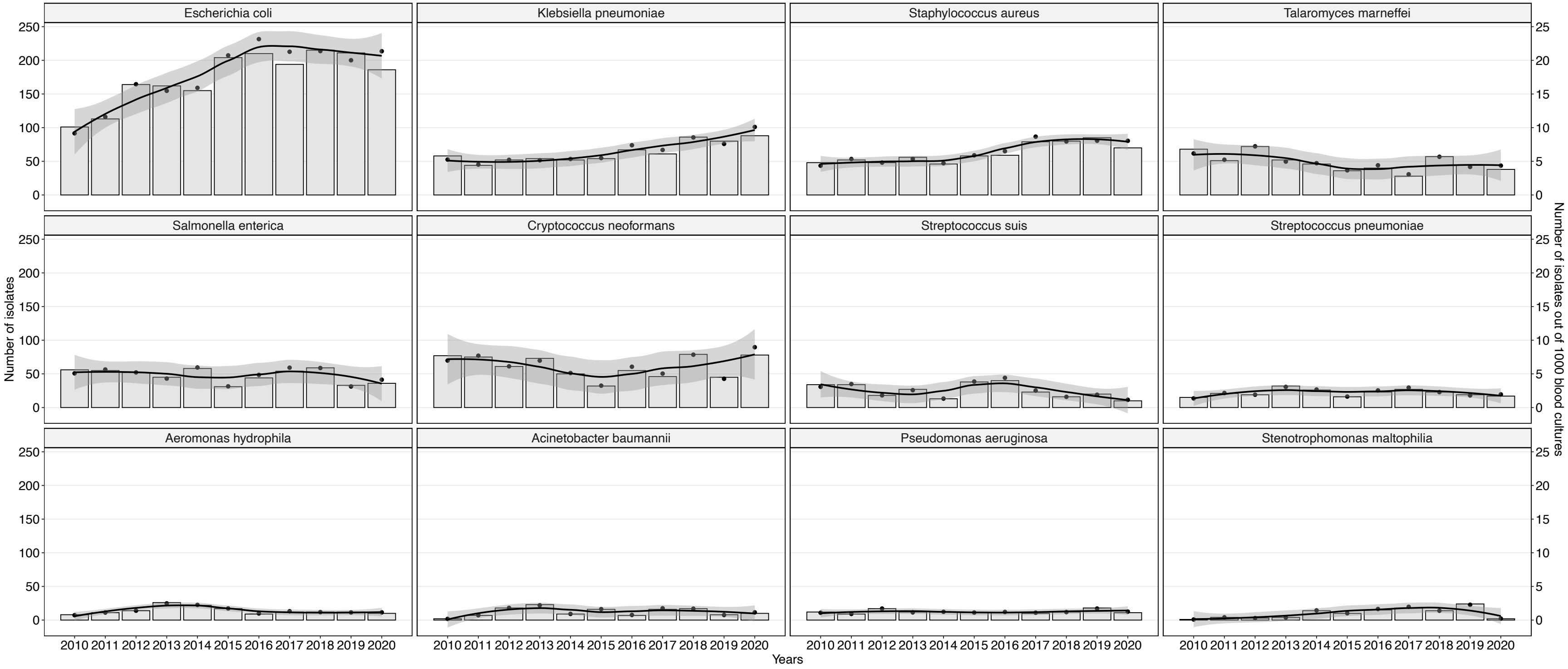

Supplementary Figure 1. Trend of significant pathogens of bloodstream infections between 2010 and 2020

Each dot represents the number of bloodstream infection pathogen out of 1000 blood cultures for each year (right Y-axis). A Loess smoothing function creates a smoothed curve through the data points, depicting the temporal trend of significant bloodstream infection pathogens. The shaded area indicates the standard error associated with the curve. The bar chart presents the raw number of BSI pathogens per year.

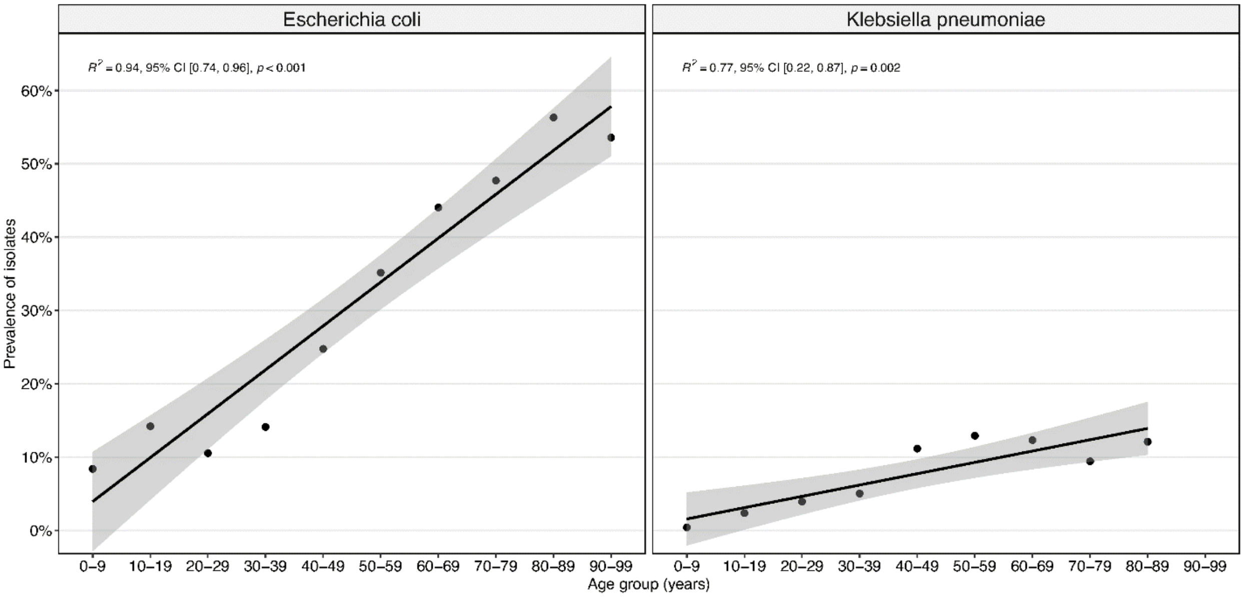

Supplementary Figure 2. Prevalence of *E. coli*/*K. pneumoniae* isolates causing community-acquired bloodstream infections by age group.

Each dot represents the proportion of *E. coli*/*K. pneumoniae* isolates causing community-acquired bloodstream infections in each respective age group. A linear regression function generates a regression line depicting the prevalence of bloodstream infections caused by *E. coli*/*K. pneumoniae* across age groups. The shaded region indicates the standard error of the linear regression.

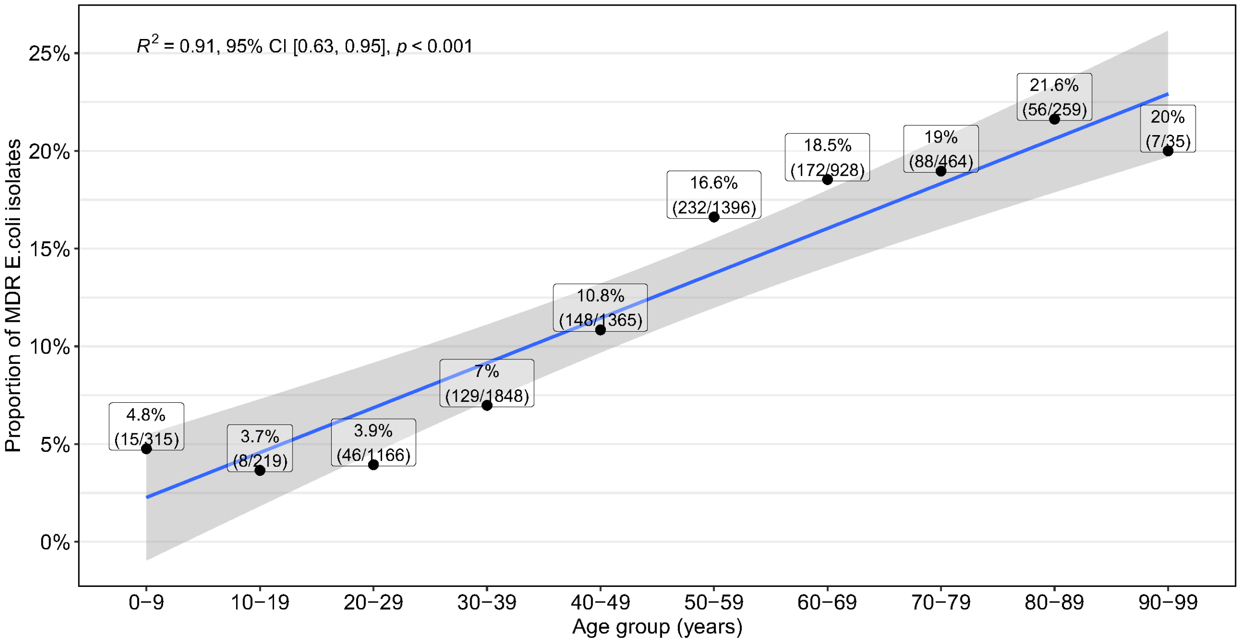

Supplementary Figure 3. Prevalence of multidrug resistant *E. coli* isolates causing community-acquired bloodstream infections by age.

Each dot represents the proportion of MDR *E. coli* isolates causing bloodstream infections in each respective age group. A linear regression function generates a regression line depicting the prevalence of bloodstream infections caused by MDR *E. coli* across age groups. The shaded region indicates the standard error of the linear regression.
